# The TNF-α antagonist etanercept reverses age-related decreases in colonic SERT expression and faecal output in mice

**DOI:** 10.1038/srep42754

**Published:** 2017-02-15

**Authors:** Bhavik Anil Patel, Sara Fidalgo, Chunfang Wang, Leena Parmar, Kasonde Mandona, Annabelle Panossian, Melanie S. Flint, Richard N. Ranson, M. Jill Saffrey, Mark S. Yeoman

**Affiliations:** 1 School of Pharmacy and Biomolecular Science, University of Brighton, Brighton, BN2 4GJ, UK; 2 Department of Life, Health and Chemical Sciences, The Open University, Walton Hall, Milton Keynes, MK7 6AA, UK; 3 Department of Applied Sciences, Faculty of Health and Life Sciences, Northumbria University, Newcastle upon Tyne, NE1 8ST, UK.

## Abstract

Treatment for chronic constipation in older people is challenging and the condition has a major impact on quality of life. A lack of understanding about the causes of this condition has hampered the development of effective treatments. 5-HT is an important pro-kinetic agent in the colon. We examined whether alterations in colonic 5-HT signalling underlie age–related changes in faecal output in mice and whether these changes were due to an increase in TNF-α. Components of the 5-HT signalling system (5-HT, 5-HIAA, SERT) and TNF-α expression were examined in the distal colon of 3, 12, 18 and 24-month old mice and faecal output and water content monitored under control conditions and following the administration of etanercept (TNF-α inhibitor; 1 mg Kg^−1^). Faecal output and water content were reduced in aged animals. Age increased mucosal 5-HT availability and TNF-α expression and decreased mucosal SERT expression and 5-HIAA. Etanercept treatment of old mice reversed these changes, suggesting that age-related changes in TNFα expression are an important regulator of mucosal 5-HT signalling and pellet output and water content in old mice. These data point to “anti-TNFα” drugs as potential treatments for age-related chronic constipation.

The incidence of chronic constipation (CC) and faecal impaction increases with age, with a prevalence of 50% in community dwelling older people rising to 74% in those that are institutionalized^1^. The treatment of CC is challenging for both the patient and clinicians and living with CC results in a reduced quality of life which may lead to a loss of independence^2^,^3^. Treatment of CC in the elderly is hampered by a lack of recruitment of older people to clinical trials and knowledge of the causes of the condition in this patient group. Previous studies have demonstrated age-related changes in colonic motility in both humans and animal models^4^,^5^,^6^,^7^,^8^, although the precise causes of these changes are currently unknown.

Changes in 5-hydroxytryptamine (5-HT) signalling can dramatically affect colonic motility and faecal output in several species^9^, although it is not clear how 5-HT signalling is affected by age. There are two main sources of 5-HT in the colon, namely the enterochromaffin (EC) cells located in the mucosa and a subpopulation of the myenteric neurons. A growing body of evidence suggests that mucosal 5-HT can act to modulate colonic motor patterns and faecal output in rodents, for review see^10^. Specifically, mucosal 5-HT can be released by both chemical and mechanical stimuli (properties of the luminal contents of the bowel), and propulsion can be attenuated by 5-HT antagonists and enhanced by 5-HT_3_/5-HT_4_ agonists applied to the mucosa or perfused intra-luminally. More recent work has exploited the observation that mucosal 5-HT is synthesized using tryptophan hydroxylase 1 (TPH1), an isoenzyme of tryptophan hydroxylase^11^ while myenteric 5-HT uses tryptophan hydroxylase 2 (TPH2)^12^. *Ex vivo* colonic motility assays from TPH1KO (TPH1 knock out) mice showed that pellet motility was slower than in wildtype mice but was overcome by increasing pellet size^13^.

In addition to impaired colonic motility, TPH1KO mice had elongated colons, reduced pellet output, increased pellet size and weight and reduced pellet water content, changes that were consistent with those we have reported previously in old mice^6^ and those observed in humans with CC^14^. Together these data strongly suggest that alterations in mucosal 5-HT can modulate colonic motility.

The strength and duration of the extracellular 5-HT signal can be exquisitely controlled by alterations in the level and activity of the serotonin transporter (SERT) which acts to clear 5-HT from the extracellular space into surrounding enterocytes where it is metabolized^15^. Altered 5-HT signalling underlies a range of GI disorders in which colonic motility is altered^16^,^17^. In many cases, these changes have been linked to changes in the expression of SERT^16^,^17^. Many of these disorders also share an inflammatory phenotype and a range of pro-inflammatory mediators e.g. tumor necrosis factor alpha (TNF-α), have been shown to down-regulate SERT expression in the colonic mucosa^18^. The ageing process is also associated with a chronic low level background inflammation in the colon of both humans^19^,^20^ and animals^21^. In the gastrointestinal (GI) tract, the causes of this low grade inflammation are unclear although changes in the gut microbiota^22^ and oxidative stress^23^,^24^ have been proposed as contributory factors. Nevertheless, the link between inflammation and changes to 5-HT signalling raises the potential for anti-inflammatory drugs to also have beneficial effects on 5-HT signalling and colonic motility.

Our study focused on an examination of the distal colon of the mice as we had previously shown that this region of the lower bowel contributed significantly to the reduced colonic motility in aged animals^6^. The current study tested the hypothesis that treatment of aged mice with the TNF-α antagonist, etanercept could attenuate the age-related decrease in pellet output and water content in mice and examined whether these changes could be explained by alterations in colonic 5-HT signalling processes.

## Results

### Age-related changes in 5-HT content and availability in the distal colon

**Figure 1:**
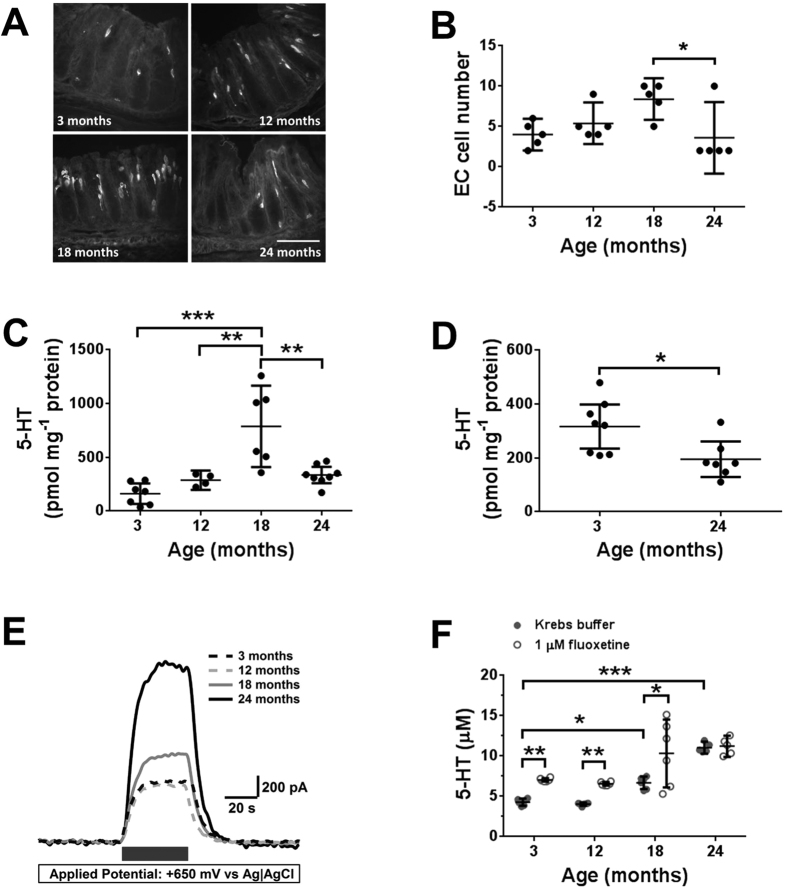
Effects of age on 5-HT levels in the mouse distal colon. (**A**) Photomicrographs of 10 μm sections showing the mucosa of the distal colon from 3, 12, 18 and 24-month tissue. EC cells labelled with an anti-5-HT antibody and a fluorescent secondary antibody are shown as white on these black and white images. Scatter plots show (**B**) the mean number of EC cells (n = 5) (**C**) Mucosal 5-HT levels (n =≥ 6) and (**D**) myenteric 5-HT levels (n =≥ 7). (**E**) Oxidation traces obtained using a boron-doped diamond electrode held at +0.65 V. With the electrode held away from the tissue (beginning of trace) the 5-HT oxidation current is low. With the electrode 0.1 mm from the tissue (grey bar) the current increases rapidly and plateaus within 20 s. Removal of the electrode from the tissue (end of trace) reduces the oxidation current to baseline. (**F**) Scatter plot showing the increase in 5-HT oxidation current with increasing age (p < 0.0001) in the presence and absence of fluoxetine (n = 6). *p < 0.05, **p < 0.01, ***p < 0.001. Data show the mean ± SD.

A combination of immunohistochemistry and high performance liquid chromatography (HPLC) were used to examine the effects of age on the intracellular 5-HT content of the distal colon. Numbers of EC cells increased steadily between 3 and 18 months and then decreased significantly between 18 and 24 months (Fig. 1A,B). There was no significant difference between the numbers of EC cells in the 3 and 24 month groups. Age-related changes in mucosal 5-HT content showed a similar trend to the change in EC cell number (Fig. 1C) consistent with the EC cells being the main source of 5-HT in the mucosa. Myenteric 5-HT levels were significantly decreased at 24-months compared to 3 months (Fig. 1D).

5-HT has to be released in order to affect the function of its targets in the colon. We were therefore interested in examining the extracellular level of 5-HT (5-HT availability) and how this changed with age. We chose to utilize constant potential amperometry to measure extracellular 5-HT as this technique provides a method to observe real time changes in the levels of oxidizable substances such as 5-HT. Our initial study focused on mucosal 5-HT. Differential pulse voltammetry performed on 3 month distal colon demonstrated a clear oxidation peak at +600 mV that was consistent with the oxidation of 5-HT (see supplementary Fig. S1). Previous studies have demonstrated that 5-HT is the only substance released from the mucosa that is oxidized at +650 mV^25^,^26^,^27^, indicating that the oxidation signals presented in this paper are solely due to the oxidation of 5-HT.

Using constant potential amperometry we demonstrated that increasing age increased mucosal 5-HT availability (Fig. 1E,F). Post-hoc analysis demonstrated a significant increase in 5-HT availability in both the 18-month and 24-month tissue compared to 3-month controls. 5-HT availability at the electrochemical sensor is dictated by a combination of the amount of 5-HT released from the tissue, its rate of re-uptake via the serotonin transporter (SERT) and also by diffusion. There were no clear changes in release with increasing age (data not shown) so subsequent figures have focused on the involvement of age-related changes in re-uptake via SERT. In order to examine the involvement of SERT in the age-related increase in 5-HT availability, we utilized fluoxetine, a potent inhibitor of SERT. If SERT was functional, then blockade with fluoxetine would inhibit re-uptake increasing the 5-HT signal at the sensor. Application of fluoxetine caused a significant increase in 5-HT availability in 3, 12 and 18-month colon but failed to alter 5-HT availability in 24-month colon (Fig. 1F). These data are consistent with SERT being functional in the three younger groups, but not in the 24-month colon.

**Figure 2:**
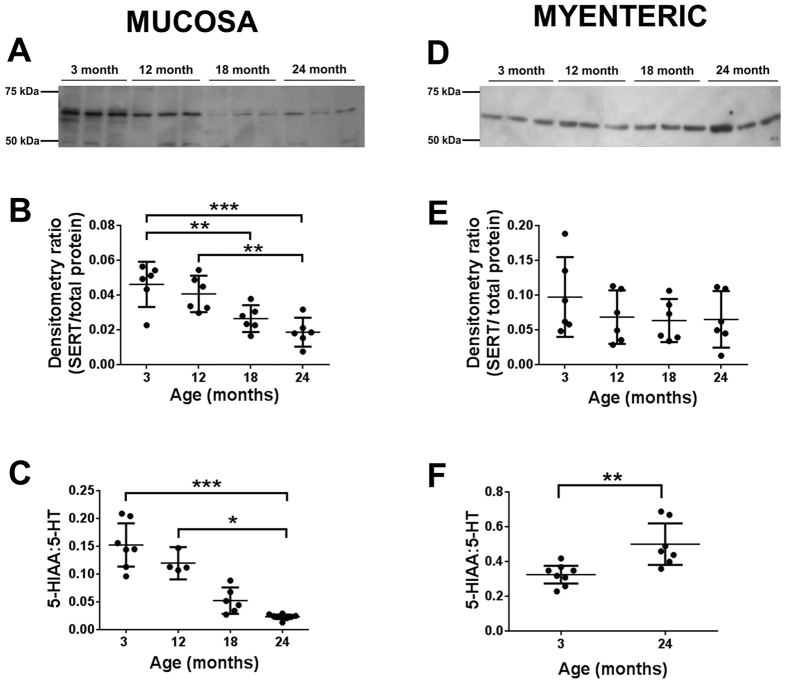
Effects of age on SERT expression and the 5-HIAA:5-HT ratio in the mouse distal colon. The anti-SERT antibody detected a major band at approximately 67 kDa in both the mucosa (**A**) and the myenteric preparation (**D**). (**B**) Normalisation of the bands to the total protein load of the labelled membrane showed that the density of the bands in the mucosa was greater in the young compared to the old group (n = 6). (**C**) Scatter plot showing the effects of age on mucosal 5-HIAA:5-HT ratio (n = 4–7 per group). (**E**) Scatter plot showing that age has no effect on myenteric SERT expression (n = 6). (**F**) Age does not affect myenteric 5-HIAA levels (n = 7–8 per group). *p < 0.05, **p < 0.01, ***p < 0.001 Data plotted represent the mean ± SD. Western blot shown are cropped gel blots.

We were unable to measure 5-HT availability in the myenteric plexus. This does not appear to be due to dramatic differences in the levels of 5-HT (compare Fig. 1C,D) or SERT (compare Fig. 2B,E) in this tissue, which are comparable to those in the mucosa, but most likely represents the location of the release sites in the myenteric preparation which lie deep within the muscle inhibiting 5-HT diffusion to the sensor.

### Effects of age on SERT expression and 5-hydroxyindoleacetic acid (5-HIAA) levels in mouse distal colon

To further quantify how the expression of SERT was altered with age we analysed colonic samples from the four age groups of mice with Western blot. Using a SERT-specific antibody a single major protein band with a molecular weight of approximately 67 kDa was identified in all age groups in both the mucosa (Fig. 2A) and the myenteric preparations (Fig. 2D), equivalent to the molecular weight of SERT identified previously in the mouse^28^. In the mucosa, the intensity of the normalised band decreased with increasing age (Fig. 2A,B), confirming a reduction in the expression of SERT with increasing age. 5-HIAA is the major metabolite of 5-HT and is formed following the re-uptake of 5-HT into enterocytes via SERT. The ratio of mucosal 5-HIAA:5-HT, was significantly decreased in 24-month mucosa compared to both 3 and 12-month groups, consistent with the reduction in SERT expression (Fig. 2C).

In the myenteric samples, SERT expression was unaffected by age (Fig. 2D,E). However, the 5-HIAA:5-HT ratio was increased in the 24 month group (Fig. 2F). This was not due to a change in the level of 5-HIAA (consistent with the lack of change in SERT, data not shown) but was due to the age-related decrease in myenteric 5-HT (Fig. 1D).

### Effects of age on TNFα levels in the distal colon

**Figure 3:**
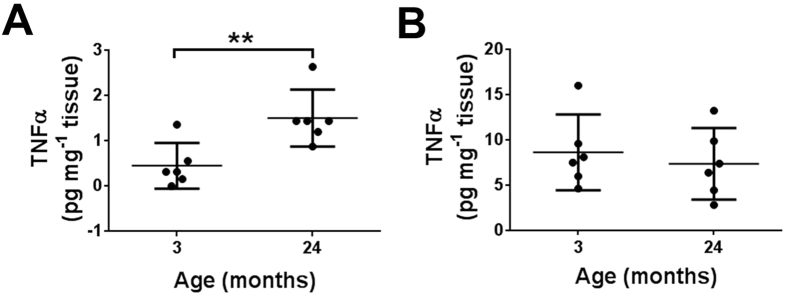
Effects of age on TNFα expression in the distal colon. Increasing age is associated with an increase in mucosal TNFα expression (**A**) but not myenteric TNFα expression (**B**). Values are mean ± SD, n = 6 per group. **p < 0.01.

Increasing age is associated with low-level chronic inflammation in many tissues including the colon, and pro-inflammatory mediators such as TNF-α have been shown to down-regulate SERT expression in the colonic mucosa^18^. We therefore examined if TNF-α levels in the distal colon changed with age in the mouse using an ELISA based assay and focused our attentions on the extremes of age (3 and 24 months). TNF-α levels were significantly higher in the distal colon mucosa of 24-month animals compared to 3-month controls (Fig. 3A). Distal colonic myenteric levels of TNF-α were unaffected by age but overall were approximately 8 fold higher than in the mucosa (Fig. 3B).

### Effects of age and the TNFα inhibitor etanercept on pellet output and water content

**Figure 4:**
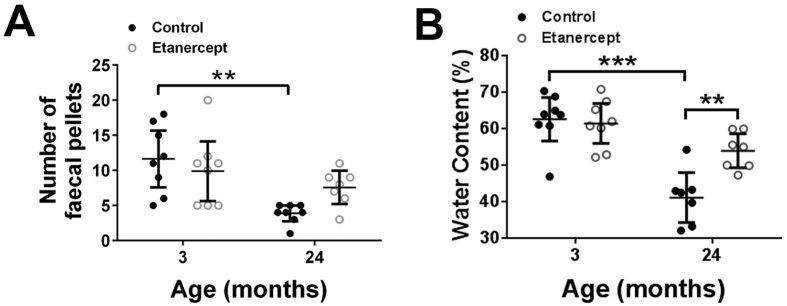
Effects of etanercept treatment on pellet output and water content. Faecal pellet output (**A**) and water content (**B**) are reduced with age and reversed in 24-month animals following etanercept treatment (n = 7–8 per group). Etanercept has no effect on pellet output and water content in 3-month animals. Values are mean ± SD, n = 6 per group. **p < 0.01; ***p < 0.001.

To understand if the observed increase in mucosal TNF-α was responsible for the age-related changes in faecal output in mice, we treated 3 and 24-month old mice with the TNF-α antagonist, etanercept. In untreated mice, the number of faecal pellets (Fig. 4A) and their water content (Fig. 4B) were significantly reduced in mice aged 24-months compared to 3-month controls. Strikingly, 24-month old animals treated with etanercept demonstrated a doubling of pellet number compared to age matched controls and a significant increase in the water content of the faecal pellets (Fig. 4A,B). Furthermore, pellet number and water content in the etanercept treated 24-month old animals were not significantly different to 3-month controls demonstrating the ability of entanercept to reverse the age-related functional changes.

### Effects of etanercept on 5-HT signalling in distal colon

**Figure 5:**
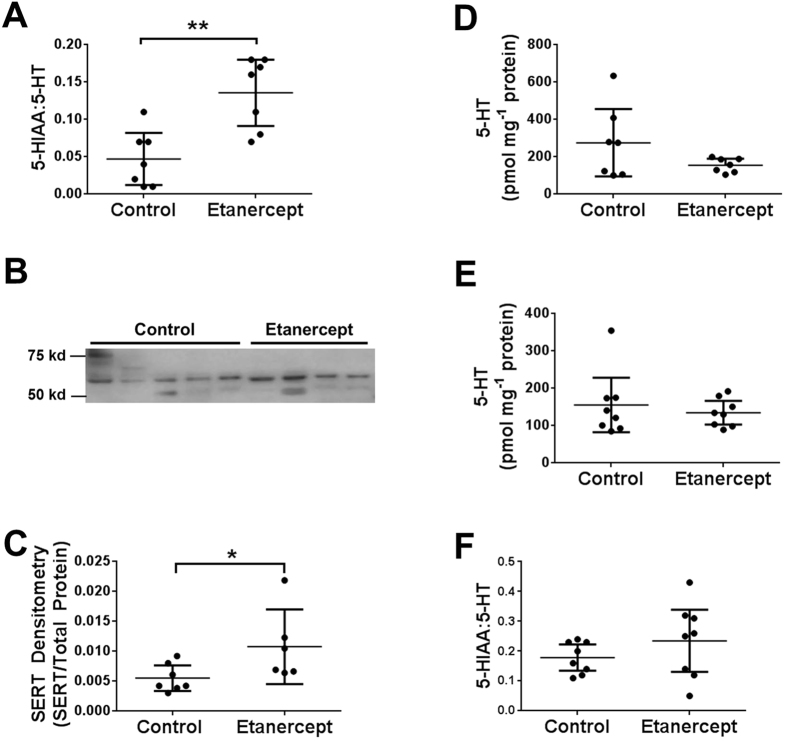
Pre-treatment with etanercept reverses age-related changes in mucosal SERT expression and the 5-HIAA:5-HT ratio . Etanercept increases mucosal 5-HIAA:5-HT ratio (**A**) and mucosal SERT expression in 24-month colon (**B**/**C**) n = 7 per group). Etanercept treatment has no significant effect on mucosal 5-HT levels in 24 month colon (**D**; n = 7) or 3 month tissue (**E**; n = 8) or on the 5-HIAA:5-HT ratio in 3-month animals (**F**; n = 8). *p < 0.05, **p < 0.01. Values are mean ± SD. Western blot shown are cropped gel blots.

As age affected components of both mucosal and myenteric 5-HT signalling and because both components have previously been shown to be important in regulating colonic motility, we were interested to examine which component of 5-HT signalling was affected by etanercept. Etanercept increased the mucosal 5-HIAA:5-HT ratio (Fig. 5A) and SERT expression in the 24-month old distal colon (Fig. 5B,C), consistent with it reversing the effects of increased age. However, etanercept had no direct effect on mucosal 5-HT levels (Fig. 5D).

We next examined whether etanercept treatment could alter 5-HT signalling in the myenteric preparation, as we had previously demonstrated that increasing age reduced myenteric 5-HT levels and that myenteric TNF-α levels were much higher than those in the mucosa. Interestingly, levels of 5-HT in 24 month myenteric preparations were unaffected by etanercept (see supplementary Fig. S2A). However, etanercept did reduce the 5-HIAA:5-HT ratio in the 24 month myenteric preparation (see supplementary Fig. S2B), consistent with it reversing the effects of age. However, these changes occurred in the absence of any changes in SERT expression and 5-HT levels, suggesting that etanercept is most likely reducing 5-HIAA levels either though an increase in the function of SERT or through an increase in the activity of monoamine oxidase A.

In order to determine whether the effects of etanercept were specific to its ability to bind TNF-α we examined whether etanercept affected 5-HT signalling in the distal colon of 3-month control mice that had negligible levels of TNF-α. Etanercept had no effect on mucosal (Fig. 5E,F) or myenteric (see supplementary Fig. S2C,D) 5-HT levels or the 5-HIAA:5-HT ratio in 3-month treated animals.

## Discussion

The main findings of this study are 1) TNF-α expression is elevated in the distal colon of 24 month old mice and this change is associated with a decrease in faecal pellet output and pellet water content; 2) Treatment of 24-month old mice with etanercept, a TNF-α antagonist, reversed the age-related decreases in pellet output and water content; 3) Etanercept reversed the age-related increase in mucosal 5-HT availability and decrease in SERT expression suggesting that the age-related increase in TNF-α observed in this tissue is responsible for the changes in SERT expression.

Work in this and a previous study^6^ has demonstrated that aged mice develop a phenotype that has some of the characteristics of human constipation (reduced faecal output and water content). We have extended our previous study to understand further the underlying causes of these changes.

**Figure 6:**
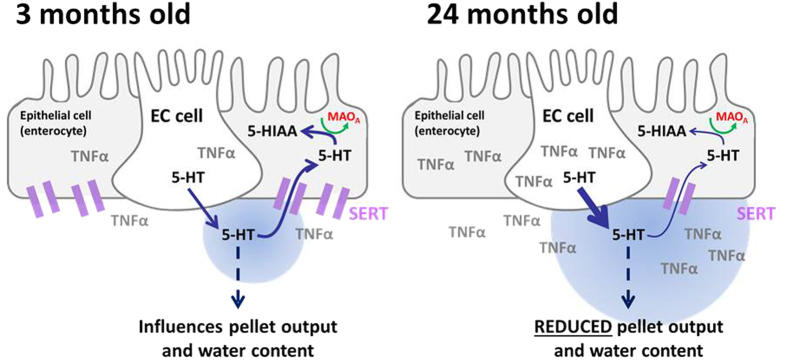
Diagrammatic representation of the putative mechanisms regulating pellet output and pellet water content in the mouse.

As 5-HT is a major regulator of colonic motility and therefore faecal output we examined how mucosal and myenteric 5-HT signalling changed with age. Age increased distal colonic mucosal 5-HT availability through a reduction in SERT expression. Consistent with this, was the observation that the mucosal 5-HIAA:5-HT ratio was also reduced with age, as the metabolism of 5-HT to 5-HIAA requires the re-uptake of 5-HT into the cytoplasm via SERT (Fig. 6). There is a paucity of data examining how mucosal 5-HT signalling changes in older humans with chronic constipation. Lincoln *et al*.^29^ showed a significant increase in mucosal 5-HT in patients with idiopathic constipation^29^. In a more recent study, Costedio *et al*.^30^ demonstrated an increase in mucosal 5-HT availability that was shown to be due to an increase in EC cell number, 5-HT content and 5-HT release in the absence of changes in SERT expression^30^. Both studies in humans are consistent with the increased 5-HT availability reported in this current study. However, the increased EC cell number and 5-HT content observed in both the human studies is more representative of the changes we observed in the 18-month colon samples rather than the oldest 24-month group. One possible explanation for this discrepancy is that the observed changes may be reflective of the relatively low mean age of the participants in the study by Costedio *et al*. (24–74 years, mean age 42 years). Indeed, Mawe and Hoffmann have recently suggested that the increased EC cell numbers and mucosal 5-HT content associated with coeliac disease, compared to the reduced numbers of EC cells and 5-HT content observed in inflammatory bowel disease (IBD) was a function of the duration of the inflammatory condition with IBD being longer than coeliac disease^10^. These suggestions are interesting and might provide an explanation for our observed increase in EC cells number and mucosal 5-HT content in mice between 3–18 months and the subsequent decrease in both parameters between 18 and 24 months. Age-related increases in both distal colon EC cell number and expression of the isoform of tryptophan hydroxylase present in the EC cells (TPH1) have also been reported by Keating *et al*.^31^. However, their changes were observed at 24 months compared to 18 months in our current study^31^. This time discrepancy may reflect differing levels of inflammation in the colon due to different housing conditions or differences in the backgrounds of the two strains of C57BL/6 mice. Inflammatory mediators have previously been shown to post-translationally regulate SERT expression^32^ and this could explain why Keating *et al*. saw no difference in SERT mRNA expression with age but we observed clear decreases in SERT protein expression at both 18 and 24 months.

The most interesting finding from this study was that treatment of mice with etanercept for 2 weeks was capable of attenuating the age-related changes in pellet output and water content, mucosal SERT expression and 5-HIAA:5-HT ratio. Previous studies have shown that pro-inflammatory mediators such as TNF-α can reduce SERT expression in the colonic mucosa in rodent models of inflammatory bowel conditions^18^,^33^,^34^,^35^,^36^,^37^ observations that are consistent with our findings. Despite the clear reversal of the aged phenotype in the distal colon mucosa, etanercept failed to reverse the age-related decrease in myenteric 5-HT.

If age-related changes in pellet output and water content can be reversed by etanercept and etenercept binds free TNF-α, then what are the main drivers for the increase in mucosal TNF-α? Previous studies using acute applications of dextran sulfate sodium or Trinitrobenzenesulfonic acid to the colon of young mice have demonstrated an increase in mucosal TNF-α and this increase is inhibited in TPH1KO mice or by drugs that selectively inhibit mucosal 5-HT synthesis^38^,^39^. These observations suggest that the age-related increase in 5-HT overflow could be driving the increase in TNF-α. However, as TNF-α has been shown to decrease mucosal SERT expression^18^, and would therefore increase mucosal 5-HT overflow, it is possible that the increase in mucosal TNF-α could occur independently of 5-HT. Interrupting this pathway using etanercept to block TNF-α signalling increased mucosal SERT expression and the 5-HIAA:5-HT ratio, without altering mucosal 5-HT, suggesting that this pathway was at least partially responsible for the age-related changes in mucosal 5-HT overflow. Together these findings suggest that while mucosal 5-HT may contribute to the increase in mucosal TNF-α with age other age-related changes in the colon may also be involved. These alternative causes of the increase in mucosal TNF-α are currently unknown but are under investigation. Potential contributing factors include an increase in replicative senescence and the development of a pro-inflammatory senescence associated secretory phenotype (SASP)^40^. Alternatively, inflammation due to activation of the immune system^21^,^41^, reduced colonic polyamine levels^42^, as a consequence of altered gut microbiota^22^ may also contribute^43^,^44^. Most recently, vitamin D deficiency, a common hallmark associated with ageing, has been demonstrated to induce colonic inflammation through the secretion of senescence-associated inflammatory cytokines^45^.

If 5-HT is a pro-kinetic agent, then why does an increase in mucosal 5-HT availability impair faecal output? Previous studies have demonstrated that pharmacological blockade of mucosal SERT and a consequential increase in 5-HT availability impairs colonic motility and faecal output in both humans and rodent models^46^,^47^. The authors proposed that this was due to the high levels of mucosal 5-HT which lead to desensitization of 5-HT_3_ receptors on intrinsic primary afferent neurons impairing the peristaltic reflex. Treatment with etanercept was able to reverse the effects of age on both mucosal 5-HT availability and faecal output, strongly suggesting that the age-related change in mucosal 5-HT availability was a major contributor to impaired faecal output in aged mice. Indeed TPH1KO mice, in which mucosal 5-HT levels are dramatically reduced, also show impaired colonic motility in *ex vivo* assays confirming a role for mucosal 5-HT in regulating colonic motility^13^. Interestingly, this model also demonstrated other features in common with our aged phenotype namely an elongated colon and faecal impaction^6^ further supporting a role for disrupted mucosal 5-HT signalling in the aged phenotype. However, earlier studies comparing both TPH1KO and TPH2KO mouse showed that colonic motility was only impaired in the TPH2KO mouse in which myenteric 5-HT was significantly reduced^48^. The discrepancy between these two studies most likely represents the different methods used to assess colonic motility and it is likely that a combination of both mucosal and myenteric 5-HT release are required for appropriate colonic motility in young animals^49^. While our aged phenotype could be explained by an impairment in mucosal 5-HT signalling, we did observe a reduction in myenteric 5-HT suggesting that neuronal signalling was also altered with age. However, myenteric TNF-α expression was not altered with age and the effects of age were not reversed by etanercept suggesting that this is not the main mechanism by which age or etanercept have their effects on motility. Although there were no age-related changes in myenteric TNF-α levels, it was interesting to note that treatment with the TNF-α antagonist etanercept decreased myenteric 5-HIAA:5-HT ratio potentially providing a compensatory increase in myenteric 5-HT. Based on these findings we cannot exclude the possibility that a component of the ability of etanercept to rescue the aged-phenotype is through its actions on neuronal 5-HT signalling.

Previous studies have also shown that TNF-α can have a range of direct effects on colonic tissue providing the possibility that etanercept can directly influence faecal output and water content. However, TNF-α suppresses fluid absorption and favours secretion of fluid into the lumen of the bowel^50^,^51^. Therefore etanercept treatment would be predicted to reduce pellet water content and this would not explain the increase we observed. However, fluid absorption from the colon is also affected by colonic transit time, with slower transit times increasing fluid absorption. TNF-α has been shown to reduce the strength of smooth muscle contractions in mice^52^,^53^, through a mechanism that involves a decrease in C-kinase-activated protein phosphatase-1 (PP1) inhibitor, 17 kDa (CPI-17) levels. This would be expected to slow colonic transit and increase fluid absorption. However, the lack of change in TNF-α in the myenteric/muscle preparation with age suggests that the ability of TNF-α to inhibit colonic muscle contractions is likely to have only a minimal effect on colonic motility in our aged animals.

The data presented here suggest that etanercept and related TNF-α antagonists could be useful treatments for age-related CC. However, work on higher organisms is inconclusive as to whether colonic TNF-α levels increase with age. A study in baboons >18 years old, showed that TNF-α levels are not increased with age^54^. However, baboons can live to 45 years old in captivity, suggesting that a number of these animals had barely reached middle age. Studies on humans are also inconclusive; with some documenting age-related increases in TNF-α^55^ while others do not^56^. Interestingly, mild diarhoea is a common side effect of patients taking etanercept for their rheumatoid arthritis although how much of this is due to a concomitant reduction in the use of opioid based pain killers is unclear.

The potential to utilize TNFα antagonists to treat age-related constipation in humans is not without its problems. First, the cost of the drugs is likely to make treatment prohibitively expensive, making it important to elucidate the optimum dose and dosing frequency in animal models before trialing the treatment in humans. There is also the potential issue that anti-inflammatory drugs, like etanercept, that suppress the immune system may increase the incidence of infections in older people whose immune system is suppressed. However, in a study of the long term safety of etanercept in the elderly population the incidence of adverse events, serious adverse events, infectious events, medically important infections, and malignancies was not significantly raised in elderly subjects in comparison with subjects less than 65 years old^57^. Finally, etanercept and all other clinically available TNF-α antagonists are administered by injection, which may well reduce compliance. Should these drugs prove efficacious in humans for the treatment of age-related constipation, then industry should be encouraged to develop orally active TNF-α antagonists that do not cross into the systemic circulation.

Finally, given the results from this and other studies that have examined the role of colonic 5-HT signalling on motility, it is tempting to infer that the effects of etanercept are through its ability to restore age-related changes in both mucosal and myenteric 5-HT signalling. Given the complexity of colonic 5-HT signalling processes and the changes we have observed with age, directly demonstrating the link between etanercept treatment, 5-HT signalling and improved bowel movements is at best difficult. Our main finding is that mucosal SERT expression is down-regulated with age. Attempts to mimic this change by administering the SERT antagonist, fluoxetine, to young animals may well inhibit faecal output and water content but whether these effects are due to changes in mucosal 5-HT signalling or changes in myenteric or CNS 5-HT signalling processes will be unclear. Additionally, analyzing the effects of increasing TNF-α levels in young animals to try to mimic the effects of age on this pro-inflammatory mediator would also be unclear because the changes we have observed in TNF-α expression are very region specific and whole animal dosing will not generate similar region specific changes. Therefore in the absence of a conclusive set of experiments the link between etanercept treatments, alterations in mucosal/myenteric 5-HT signalling and age-related changes in pellet output remain inconclusive.

These data demonstrate the reversal of an age-related functional phenotype in the lower bowel with an anti-inflammatory drug. This work provides an exciting insight into one of the potential causes of age-related colonic dysfunction in mice. The observation that the TNF-α antagonist etanercept can reverse age-related changes in mucosal SERT expression, 5-HIAA levels and the decrease in faecal pellet output and water content highlights the potential for “anti-TNFα” drugs to alleviate age-related colonic dysfunction. In the short term, efficacy in humans may be established using existing drugs, but longer-term this provides an opportunity to develop GI tract specific analogues of etanercept to treat colonic dysfunction.

## Methods

### Ethical approval

All procedures were carried out according to U.K. Home Office regulations and the Animal [Scientific Procedures] Act 1986) and were approved by the University of Brighton Ethics Committee. The manuscript was prepared according to the ARRIVE guidelines.

### Animals

8 week old male C57BL/6 J mice (Harlan UK) were aged in individual ventilated cages under barrier-reared conditions. Animals were maintained at 19.0 ± 1 °C, 55% humidity and fed on a maintenance diet (RM1 (E) 801002 (Special Diet Services) chow). Initial studies were performed on 3, 12, 18 and 24 month old animals to characterize the time course of changes in 5-HT signalling. Later experiments focused on 3 and 24 month old mice to understand whether inflammation was a cause of the observed functional and biochemical changes.

### Administration of Etanercept

Etanercept (1 mg Kg^−1^) was administered intraperitoneally (i.p.) twice a week for 2 weeks. Control animals were injected i.p. with sterile water.

### Monitoring of faecal pellet output

Faecal pellet output was performed in an arena divided into three equal sized sections (14 × 25 cm) by perforated Perspex dividers. Individual mice were placed in each section so they could visualise and smell their cage mates. Pellets were collected for 1 hour and their number and total wet weight determined. Pellets were dried overnight at 50 °C and their dry weight and water content determined.

### Intestinal preparation

Animals were asphyxiated with CO_2_ and exsanguinated following cervical dislocation. The whole colon was harvested 1 cm proximal to the anus and placed in oxygenated (95% O_2_ and 5% CO_2_) Krebs buffer solution, pH 7.4 (117 mM NaCl, 4.7 mM KCl, 2.5 mM CaCl_2_, 1.2 mM MgCl_2_, 1.2 mM NaH_2_PO_4_, 25 mM NaHCO_3_ and 11 mM glucose). Based on our previous findings that *ex vivo* colonic motility was impaired with age and that this impairment was due solely to a reduction in motility in the distal colon^6^ we have focused our investigations in this current study on 5-HT signalling in the distal colon. The distal colon was identified as the distal most half of the colon and used for the experiments described below.

### Amperometric monitoring of 5-HT availability

Amperometric measurements were made using a boron-doped diamond (BDD) microelectrode as previously described^58^. Tissue samples were perfused with warm (37 °C) Krebs solution at a flow rate of 2 mL/min. Continuous amperometric recordings of 5-HT overflow were carried out using a BioStat™ multi-channel potentiostat (ESA Biosciences, Inc, USA) with the electrode potential held at +650 mV vs Ag|AgCl. Briefly, using a micromanipulator, the BDD electrode was positioned several centimetres away from the mucosa for several seconds to obtain a baseline reading. To record mucosal 5-HT availability, the electrode was advanced and positioned 0.1 mm from mucosal surface for 40 s; at this distance 5-HT oxidation currents were reproducibly recorded. This procedure was repeated 5 times for each tissue. In a number of experiments the effects of the SERT antagonist, fluoxetine on 5-HT overflow was determined in all four age groups. Briefly, following a set on control readings in Krebs buffer solution the tissue was perfused for 20 minutes with 1 μM fluoxetine and 5-HT availability determined as described above.

### HPLC measurements of distal colonic 5-HT and 5-HIAA levels

The mucosa was removed from 1 cm^2^ segments of the distal colon from 3, 12, 18 and 24 month old animals using a sharp scalpel and this and the remaining tissue, consisting of the longitudinal and circular muscle layers and the myenteric plexus (subsequently called the myenteric sample) were placed separately in 500 μl of ice cold 0.1 M perchloric acid. Samples were homogenised and centrifuged at 14,600 g for 10 minutes prior to chromatographic analysis. HPLC analysis of 5-HIAA and 5-HT was then carried out according to the method of Parmer *et al*.^59^.

### SERT Western Blot Analysis

The mucosa/myenteric samples from 2 cm segments of distal colon were isolated and snap frozen in liquid N_2_ for storage. Tissue was homogenized on ice in lysis buffer (10 mM HEPES, 150 mM NaCl, 1 mM EDTA, 0.2% Nonidet P40, protease inhibitor cocktail P8340, Sigma-Aldrich Inc.). Lysates were centrifuged and supernatants were removed and their protein content assessed using Quick Start Bradford Dye reagent (Bio-Rad)^60^. 15 μg of protein from each sample was separated using SDS-PAGE. Antibodies were diluted in Tris-buffered saline + 0.1% Tween (TBST). Membranes were incubated overnight at 4 °C with a rabbit anti-SERT antibody (#24330, Immunostar Inc; 1:1500 in TBST) followed by a goat anti-rabbit HRP-conjugated secondary antibody (1:2000 in TBST, SC-2005, Santa Cruz Biotechnology) for 1 hr at room temperature. Protein bands were detected using ECL plus western blotting detection system (GE Healthcare). The relative intensity of each SERT band was normalised to the total protein load determined by staining the PVDF membrane with Coomassie Brilliant Blue (Bio-Rad). This normalisation process was chosen as a number of the commonly used housekeeping genes have previously been shown to change expression with age in other tissues^61^,^62^ and there is currently a lack of proteomic data detailing the effects that age has on protein expression in the mouse colon.

### Immunofluorescence labeling of EC cells

Immunofluorescence labelling was performed on 10 μm transverse sections of distal colon. Tissues were fixed in 4% paraformaldehyde in PBS and then embedded in paraffin. Sections were deparaffinised in Histoclear and rehydrated in graded ethanol solutions. The slides were then incubated for 30 min in 2 N HCl for antigen retrieval. The sections were then incubated with 10% goat serum (S-1000; Vector lab, Peterborough, UK) for 2 hours at room temperature. Rabbit anti-serotonin primary antibody (1:8000; 20080; ImmunoStar, UK) was applied overnight at 4 °C, and then slides were washed three times with PBS (10 min each wash). Sections were incubated for 2 hours with Alexa 488 goat anti-rabbit secondary antibody (1:200, A-11034; Invitrogen, UK). After washing with PBS, sections were mounted with Fluoroshield DAPI (F6057; Sigma, UK). Sections were viewed and images were acquired using an Olympus BX-UCB microscope.

### TNFα analysis in colonic samples

TNF-α levels from mucosa/myenteric samples were determined using a R&D systems Mouse TNF-α Quantikine ELISA kit according to the manufacturer’s instructions. Levels of TNF-α were normalized to the protein content of the tissue.

### Data Analysis

For amperometic responses, current amplitudes were recorded and converted to 5-HT concentrations using calibration responses. Population means were compared using a one-way ANOVA (GraphPad Prism) and significant differences determined using post-hoc Tukey tests. In amperometric experiments where the effects of both age and fluoxetine treatment were compared, the data was analysed using a 2-way ANOVA followed by a post-hoc Tukey test. HPLC peak areas for the analytes of interest were recorded using CHI potentiostat 1050 software and converted to concentrations using calibration responses. For both these data and those comparing TNF-α expression data from 2 age groups were compared with an unpaired t-test while those comparing more than two groups were analysed using a one way ANOVA followed by a post-hoc Tukey test. Quantitative evaluation of the number of serotonin-positive cells per ten microscopic fields (x40) was assessed in four age groups. Age-related differences were determined using a one-way ANOVA (GraphPad Prism). For Western blots the ratios of the intensity of each SERT- labelled band to the Coomassie stained PVDF membrane were compared using a Kruskall Wallis ANOVA followed by a post hoc Tukey test. In experiments where the effects of age and etanercept were examined on pellet output and water content data were compared using a two-way ANOVA and Tukey post-hoc test. All analysis was carried out using GraphPad Prism Vs 6.

## Additional Information

**How to cite this article**: Patel, B. A. *et al*. The TNF-α antagonist etanercept reverses age-related decreases in colonic SERT expression and faecal output in mice. *Sci. Rep.* **7**, 42754; doi: 10.1038/srep42754 (2017).

**Publisher's note:** Springer Nature remains neutral with regard to jurisdictional claims in published maps and institutional affiliations.

## Supplementary Material

Supplementary Information
