# Supplementary material for: The TNF-α antagonist etanercept reverses age-related decreases in colonic SERT expression and faecal output in mice
Source: Sci Rep. 2017 Feb 15;7:42754. doi: 10.1038/srep42754 (PMC5309893; doi:10.1038/srep42754)

THE TNF-α ANTAGONIST ETANERCEPT REVERSES AGE-RELATED DECREASES IN COLONIC SERT EXPRESSION AND FAECAL OUTPUT IN MICE.

Bhavik Anil Patel[1], Sara Fidalgo[1], Chunfang Wang [2], Leena Parmar[1], Kasonde Mandona[1], Annabelle Panossian[1], Melanie S. Flint[1], Richard N. Ranson[3], M. Jill Saffrey[2] and Mark S. Yeoman[1]

Supplementary Figure 1: Typical voltammogram obtained using differential pulse voltammetry. With the electrode held 0.1 mm from the tissue two clear oxidation peaks can be seen at + 0.6V and + 0.75V (grey solid line), which correspond to the peaks for 5-HT (black dotted line) and melatonin (grey dotted line), respectively.

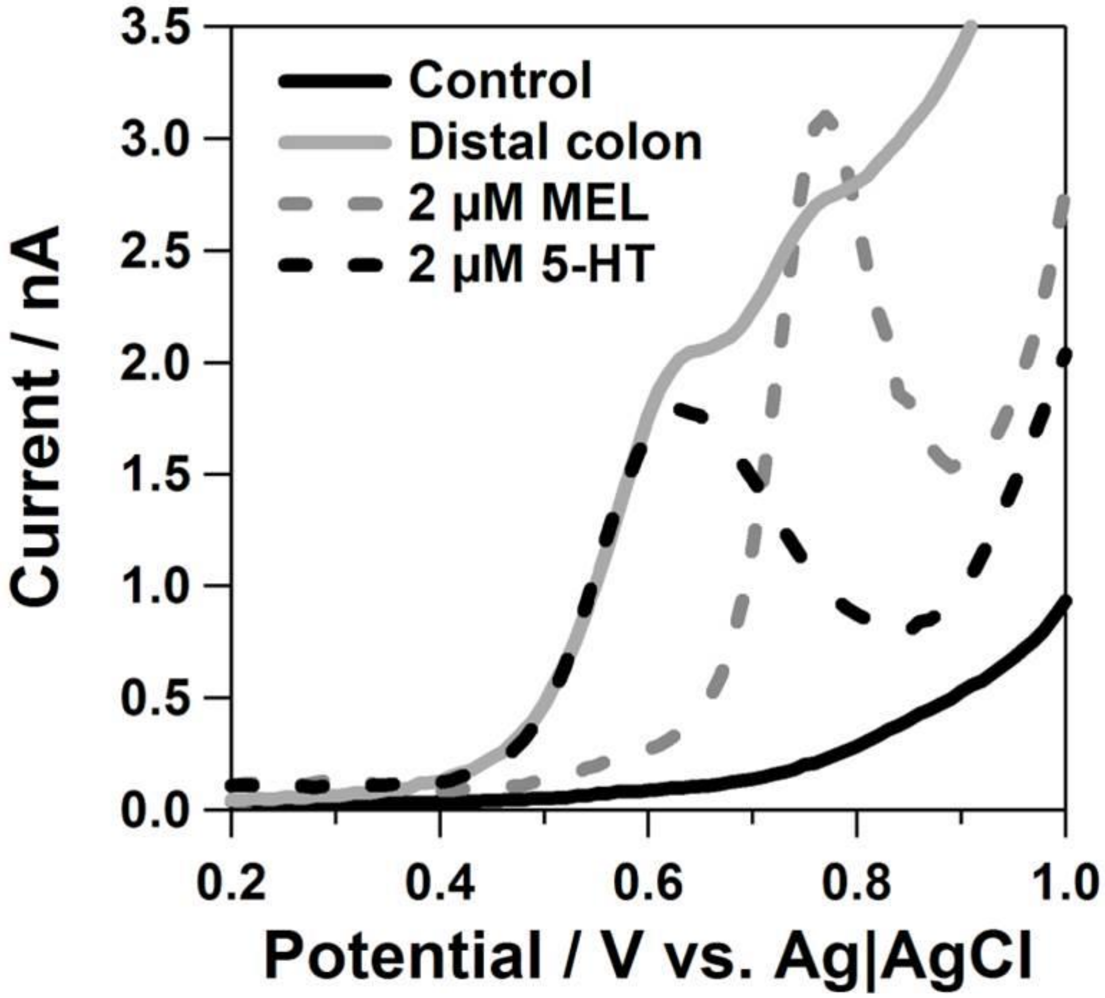

Supplementary Figure 2: Effects of Etanercept on myenteric 5-HT or the 5-HIAA:5-HT ratio in both 3 or 24 month old distal colon. In 24-month colon, etanercept had no effect on myenteric 5-HT levels (A) but reduced myenteric 5-HIAA:5-HT ratio (B). Etanercept failed to alter myenteric levels of 5-HT (C) or the 5-HIAA:5-HT ratio (D) in 3-month distal colon. * $p<0.05$, n=7 for the 24 month groups and n=8 for the 3 month groups. Data are plotted as the mean ± SD.

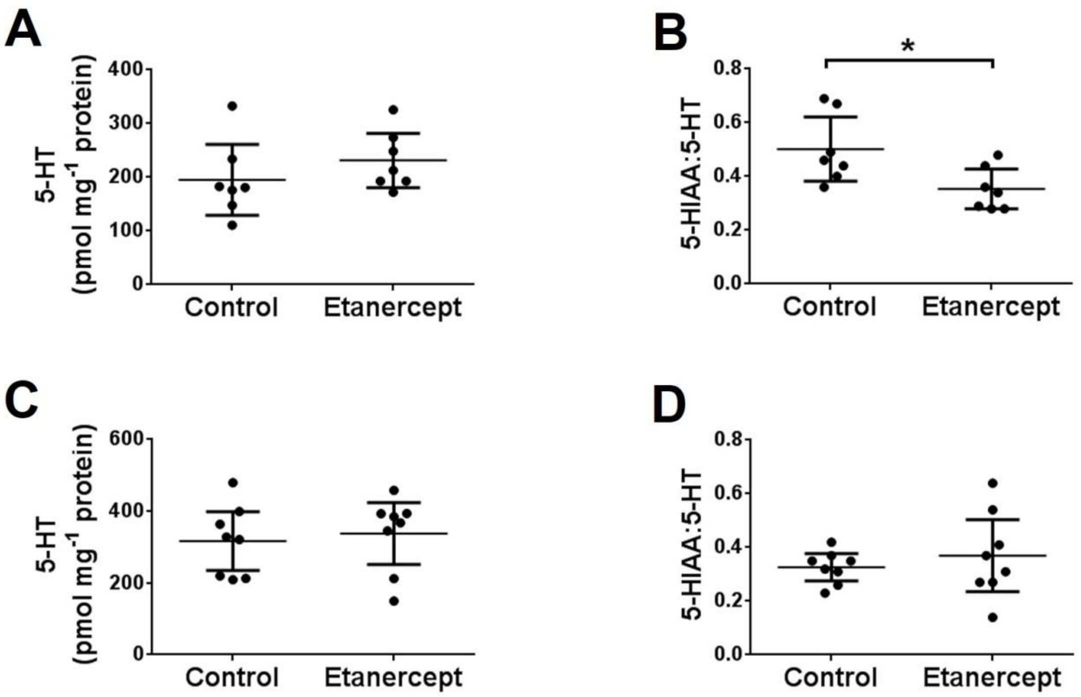

Supplement: Supplementary Information [file srep42754-s1.pdf]
